# Prevalence of Mutations in the *pfcoronin* Gene and Association with Ex Vivo Susceptibility to Common Quinoline Drugs against *Plasmodium falciparum*

**DOI:** 10.3390/pharmaceutics13081273

**Published:** 2021-08-17

**Authors:** Océane Delandre, Mathieu Gendrot, Isabelle Fonta, Joel Mosnier, Nicolas Benoit, Rémy Amalvict, Nicolas Gomez, Marylin Madamet, Bruno Pradines

**Affiliations:** 1 Unité Parasitologie et Entomologie, Département Microbiologie et Maladies Infectieuses, Institut de Recherche Biomédicale des Armées, 13005 Marseille, France; o.delandre@gmail.com (O.D.); ma.gendrot@laposte.net (M.G.); isabelle.fonta.09@gmail.com (I.F.); joelmosnier@orange.fr (J.M.); nicolas1.benoit@intradef.gouv.fr (N.B.); remy.amalvict@intradef.gouv.fr (R.A.); nicolas1.gomez@intradef.gouv.fr (N.G.); mmadamet@gmail.com (M.M.); 2 Aix Marseille University, IRD, SSA, AP-HM, VITROME, 13005 Marseille, France; 3 IHU Méditerranée Infection, 13005 Marseille, France; 4 Centre National de Référence du Paludisme, 13005 Marseille, France

**Keywords:** malaria, *Plasmodium falciparum*, antimalarial drug, *pfcoronin*, resistance, in vitro, molecular marker

## Abstract

Background: Artemisinin-based combination therapy (ACT) was recommended to treat uncomplicated falciparum malaria. Unlike the situation in Asia where resistance to ACT has been reported, artemisinin resistance has not yet emerged in Africa. However, some rare failures with ACT or patients continuing to be parasitaemic on day 3 after ACT treatment have been reported in Africa or in travellers returning from Africa. Three mutations (G50E, R100K, and E107V) in the *pfcoronin* gene could be responsible for artemisinin resistance in Africa. Methods: The aims of this study were first to determine the prevalence of mutations in the *pfcoronin* gene in African *P. falciparum* isolates by Sanger sequencing, by targeting the 874 samples collected from patients hospitalised in France after returning from endemic areas in Africa between 2018 and 2019, and secondly to evaluate their association with in vitro reduced susceptibility to standard quinoline antimalarial drugs, including chloroquine, quinine, mefloquine, desethylamodiaquine, lumefantrine, piperaquine, and pyronaridine. Results: The three mutations in the *pfcoronin* gene (50E, 100K, and 107V) were not detected in the 874 *P. falciparum* isolates. Current data show that another polymorphism (P76S) is present in many countries of West Africa (mean prevalence of 20.7%) and Central Africa (11.9%) and, rarely, in East Africa (4.2%). This mutation does not appear to be predictive of in vitro reduced susceptibility to quinolines, including artemisinin derivative partners in ACT such as amodiaquine, lumefantrine, piperaquine, pyronaridine, and mefloquine. Another mutation (V62M) was identified at low prevalence (overall prevalence of 1%). Conclusions: The 76S mutation is present in many African countries with a prevalence above 10%. It is reassuring that this mutation does not confer in vitro resistance to ACT partners.

## 1. Introduction

Since the introduction of artemisinin-based combination therapy (ACT) in 2005 [1] global malaria cases have dropped. According to the 2020 World Health Organization (WHO) report, there were 229 million malaria cases and 409,000 deaths in 2019 [2] compared to 228 million malaria cases and 405,000 deaths in 2018 [3]. However, the number of cases has been declining since 2000, from 238 million to 229 million [2]. There have been 215 million malaria cases in Africa, representing 94% of the total number of cases [2]. The use of prophylaxis for travellers in endemic areas is strongly recommended. However, and despite these recommendations, France continues to have a high rate of imported malaria, with 82,000 imported malaria cases reported between 2000 and 2015, including 2430 cases in the French Army [4,5]. During this period, most malaria cases (95%) were contracted in sub-Saharan Africa (particularly Côte d’Ivoire, Cameroon, Mali, and Senegal) [5,6]. The number of reported cases (around 50% of estimated cases) to the National Reference Centre for Malaria Surveillance peaked at 3400 cases in 2000, decreased to 1824 cases in 2005 and was around 2895 cases in 2019 [7].

Artemisinin-based combination therapy (ACT) was introduced as a result of reported resistance to other antimalarial drugs in Africa. In most African countries, artemether-lumefantrine or artesunate-amodiaquine are recommended as first-line malaria treatment, and dihydroartemisinin-piperaquine is recommended as second-line treatment in some countries [8]. Unlike the situation in Asia, and more particularly in western Cambodia, Vietnam, Myanmar, Thailand, and Laos, where resistance to ACT has been reported [9,10,11,12], artemisinin resistance has not yet emerged in Africa.

However, some rare failures with ACT or patients who remain parasitaemic on day 3 after ACT treatment have been reported in Africa or in travellers returning from Africa [13,14,15,16,17,18,19,20,21,22]. Moreover, some rare therapeutic efficacy studies (TES) reported a proportion of clinical failures on day 28 above 10% after artemether-lumefantrine treatment in Angola, the Gambia, Malawi, and Uganda [23].

In 2014, the gene coding for the kelch 13 helix (*pfk13*) was identified as a molecular marker with in vivo and in vitro association for artemisinin resistance in South-East Asia [13,24]. However, until 2020, ACT clinical failures in Africa were not associated with polymorphisms in this gene and more particularly with the mutations described in Asia [13,14,17,19,20,22,25]. Recently, a new mutation on *pfk13*, R561H, has emerged in *P. falciparum* parasites collected in Masaka (Rwanda) and has spread rapidly in Rwanda with a high prevalence of up to 22% [26,27,28]. This mutation was found to be associated with delayed parasite clearance after treatment with ACT [28,29].

In 2018, a study on Senegalese *P. falciparum* strains described three polymorphisms (G50E, R100K, and E107V) in the *pfcoronin* gene which are thought to be responsible for dihydroartemisinin resistance in Africa [30]. These mutations were identified in two culture-adapted Senegalese field isolates from Pikine and Thies (Senegal) which became resistant in vitro to artemisinin after 13 cycles of discontinued selection under dihydroartemisinin pressure for four years [30]. The role of the mutations in *pfcoronin* was confirmed by CRISPR/Cas9 editing [31]. These three mutations, initially identified in *P. falciparum* parasites after drug pressure, were not reported in isolates from Senegal, Gabon, Ghana, Congo, Kenya, or Pakistan [16,17,32,33]. A new mutation, P76S, was detected in parasites from Senegal (prevalence 16.2%), Gabon (11%), Ghana (16%), Kenya (4%), and Congo (17%) [17,32]. However, none of these mutations appears to be associated with artemisinin reduced susceptibility in field isolates [16,17]. The *pfcoronin* gene is conserved in the different species infecting humans and shows 57% identity with *Plasmodium berghei*, a rodent-infecting species [34]. This gene codes for an actin-filament binding protein involved in the motility of sporozoites of *P. falciparum* [34]. It is expressed during schizogony and is located on the periphery of merozoites before and during invasion [35].

The aims of this study were first to determine the prevalence of the mutations in the *pfcoronin* gene in African *P. falciparum* isolates, by targeting the 874 samples collected from patients hospitalised in France after returning from endemic areas in Africa between 2018 and 2019, and secondly, to evaluate their association with in vitro reduced susceptibility to standard quinoline antimalarial drugs, including chloroquine, quinine, mefloquine, desethylamodiaquine, lumefantrine, piperaquine, and pyronaridine.

## 2. Materials and Methods

### 2.1. Sample Collection

From 2018 to 2019, 874 blood samples from patients hospitalised in France after returning from endemic countries in Africa, were sent within 72 h after collection to the French National Reference Centre for Malaria Surveillance (IRBA, IHU Méditerranée Infection Marseille) by hospitals in the French National Reference Centre for Imported Malaria network. Clinical and epidemiological data were collected for each sample. The diagnosis of *P. falciparum* was determined on the basis of a stained thin blood smear and confirmed by real-time PCR [36].

### 2.2. Gene Sequencing

Analysis of *pfcoronin* (codon positions 43 to 177) was performed using the same venous blood samples used for diagnostic analysis. DNA from each sample was extracted using a QIAamp DNA Blood Kit (Qiagen, Hilden, Germany) according to the manufacturer’s recommendations.

Amplification of the *pfcoronin* gene was performed in the same way as described previously [17]. The *pfcoronin* gene (PF3D7_1251200) was amplified by PCR using the forward primer 5′-TATATCGTTTTATATGATTTGTTC-3′ and reverse primer 5′CCGATATCCCATTGTAAAGA-3′. Sanger sequencing was carried out using Biofidal (Biofidal, Vaulx en Velin, France) and the reverse primer. Sequence analysis of the samples was performed using the CodonCode Aligner software (CodonCode Corporation, Centerville, MA, USA).

### 2.3. Ex Vivo Assay

Chloroquine, quinine, mefloquine, desethylamodiaquine, lumefantrine, piperaquine, and pyronaridine were obtained from Sigma-Aldrich (St Quentin Fallavier, France). For the ex vivo chemosusceptibility assay, each isolate was aliquoted in 96-well plates previously treated with a gradient of antimalarial drug concentrations without culture adaptation, as previously described [37]. The plates were then incubated for 72 h at 37 °C in a controlled atmosphere with 10% O_2_, 5% CO_2_, and 85% N_2_. An ELISA assay was then performed by targeting the HRP2 protein using the Malaria Ag Celisa kit (ref KM2159, Cellabs PTY LDT, Brookvale, Australia) to estimate parasite growth, as previously described [38].

Each batch of plates was controlled by assessing the chloroquine-resistant *P. falciparum* strain W2 (MR4, Charlottesville, VA, USA) in between three and six independent experiments.

### 2.4. Data Management and Statistical Analysis

The IC_50_ were estimated through nonlinear regression by using ICEstimator version 1.2 [39]. Geometric means of IC_50_ values were calculated for each antimalarial drug. T-test and Chi-squared test were used to compare groups.

## 3. Results

**Figure 1:**
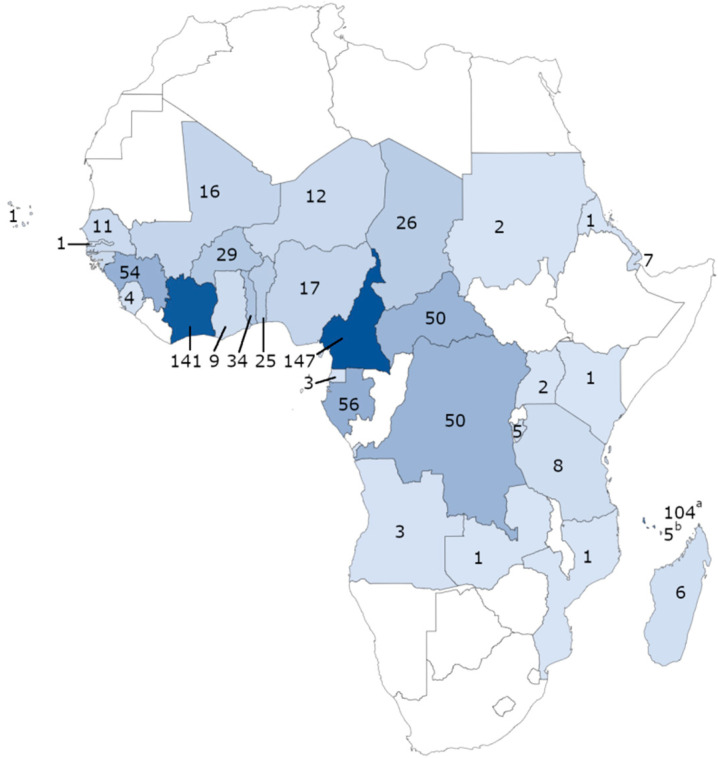
Geographical distribution and numbers of *Plasmodium falciparum* isolates collected in 2018 (397 samples) and 2019 (477 samples) from patients hospitalised in France after returning from malaria endemic areas in Africa (39 from an unspecified country in Africa) (^a^ from Comoros and ^b^ from Mayotte).

A total of 874 samples of *P. falciparum* collected from patients hospitalised in France after returning from malaria-endemic areas in Africa between 2018 and 2019, were analysed. For this study, 481 samples were analysed for 2018 and 403 samples for 2019. The samples were sent to the French National Reference Centre for Malaria Surveillance (IRBA, IHU Méditerranée Infection, Marseille) from various civilian or military hospitals in the French National Reference Centre for Imported Malaria network (Aix en Provence, Bordeaux, Lyon, Marseille, Mayotte, Montpellier, Nice, Toulon, Toulouse, and Valence). All the patients had returned from endemic areas in Africa, particularly from Cameroon (147), Côte d’Ivoire (141), Comoros (104), Gabon (56), Guinea (54), Central African Republic (50), Congo (50), Togo (34), Burkina Faso (29), Chad (26), and Benin (25) (Figure 1).

### 3.1. Analysis of pfcoronin Gene

The results of the *pfcoronin* gene sequencing on the *P. falciparum* isolates revealed the absence of the three mutations (G50E, R100K, and E107V) described previously as being associated with artemisinin resistance. From 2018 to 2019, 40.8% of the isolates studied came from West Africa, 38.3% from Central Africa, 16.1% from East Africa, and 0.2% from North Africa. Among the 874 *P. falciparum* isolates, 125 carried the P76S mutation (76S) (14.3% mean prevalence) and nine carried the V62M mutation (62M) (1% mean prevalence) (Table 1). No other mutation was identified.

The proportion of the 76S mutation was higher for patients who came from West Africa (8.5%) and Central Africa (4.5%) compared to patients from East Africa (0.7%). The 76S mutation was present in 20.7% of the isolates from West Africa, 11.9% of the isolates from Central Africa, and 4.2% of the isolates from East Africa (Table 2). The difference in the proportion of 76S mutation between the three areas was significant (*p* = 3.1 × 10^−6^; Pearson’s Chi-squared test). Concerning countries included where at least 50 *P. falciparum* isolates came from, the proportion of 76S mutation ranged from 6.0% in Congo to 24.1% in Ivory Coast (Table 2). The proportion of 76S mutation was low in East Africa (4.2%). This mutation was not detected in the 104 isolates from Comoros.

Only six patients from Central Africa (0.7%) and three patients from West Africa carried the 62M mutation (0.3%) (Table 1). This mutation was reported in Burkina Faso (6.9% of the parasites from Burkina Faso), Nigeria (5.9%), Cameroon (1.4%), Gabon (1.8), Central African Republic (4.0), and Chad (3.8%) (Table 2).

### 3.2. Drug Susceptibility

Of the 874 samples analysed by genotyping, 330 isolates were successfully assessed in vitro for at least one antimalarial drug: 328 isolates for chloroquine (CQ), 325 isolates for quinine (QN), 329 isolates for lumefantrine (LMF), 306 isolates for desethylamodiaquine (the active metabolite of amodiaquine) (DQ), 321 isolates for mefloquine (MQ), 319 isolates for pyronaridine (PND), and 321 isolates for piperaquine (PPQ). The other isolates failed in culture during the ex vivo assay or were not tested due to low parasitaemia under 0.05% or because samples were only collected as dried blood spots, such as the 104 isolates from Comoros.

The values of 50% inhibitory concentration (IC_50_) estimates ranged from 2.8 nM to 764.7 nM for CQ, 6.2 nM to 757.2 nM for QN, 0.4 nM to 68.8 nM for LMF, 0.6 nM to 775.9 nM for DQ, 0.7 nM to 98.0 nM for MQ, 0.2 nM to 119.3 nM for PND, and 0.7 nM to 379.3 nM PPQ as shown in Table 3.

### 3.3. Association between In Vitro Responses (IC50) and pfcoronin Mutations

The values of drug ex vivo susceptibility (IC_50_) of the wild-type samples (P76) (no = 281 isolates) were compared to those of the mutated parasites (76S) (no = 49 isolates) for each antimalarial drug. There was no significant difference between the two groups (Table 3) (*p*-values ranged from 0.295 to 0.983). The IC_50_ geometric means for chloroquine and mefloquine were close to their respective threshold of reduced susceptibility (100 nM and 30 nM, respectively).

Moreover, based on the cut-off values for reduced ex vivo susceptibility to CQ (100 nM), QN (800 nM), LMF (150 nM), DQ (80 nM), MQ (30 nM), DOX (35 nM), PND (60 nM), or PPQ (135 nM) [40,41], we compared the proportion of isolates with reduced susceptibility to each drug according to the genotype of *pfcoronin* (P76 or 76S). There was no significant difference between the two groups (Table 4).

## 4. Discussion

The aim of this study was to assess the prevalence of the mutations in the *pfcoronin* gene and their impact on the susceptibility to common antimalarial drugs in vitro. We did not find the three polymorphisms previously described by Demas et al. (G50E, R100K, and E107V) [30] but we identified two polymorphisms, P76S and V62M. The overall prevalence of the 76S mutation for Africa was 14.3%. We found a prevalence of 9.1% for this mutation in imported parasites from Senegal collected between 2018 and 2019. The 76S mutation had already been reported in Dakar, Senegal between 2015 and 2019 in 15.9% of the *P. falciparum* isolates [17]. More particularly, this mutation was identified in 12.5% of isolates from Dakar in 2016, 12.9% in 2017, 17.0% in 2018, and 25.9% in 2019 [17]. This mutation was not reported in parasites from the centre and the south of Senegal (Diourbel and Kedougou regions, respectively) [16].

The P76S mutation was also reported in Gabon (11%), Ghana (16%), Kenya (4%), and Congo (17%) [32]. Our data were in line with these previous observations. We found a prevalence of 8.9% in isolates from Gabon and 22.2% in isolates from Ghana.

Isolates from East Africa showed a prevalence of the 76S mutation (4.2%) which was lower than that observed in West Africa (20.7%) or Central Africa (11.9%). This mutation was not detected in the 104 isolates from Comoros.

Additionally, the 76S mutation was not reported in a study from Pakistan which analysed 179 *Plasmodium falciparum* isolates collected between 2018 and 2019 [33].

The prevalence of the 62M mutation (0.8%) was very low in Africa: 1.8% in Central Africa, 0.8% in West Africa and 0% in East Africa. This mutation was only reported in Burkina Faso (6.9%), Nigeria (5.9%), Cameroon (1.4%), Gabon (1.8), Central African Republic (4.0), and Chad (3.8%). These data were consistent with a previous study that showed an overall prevalence of 0.7% in Africa, and more particularly a prevalence of 1% in Gabon, 2% in Ghana, and 0% in Kenya and Congo [32].

It appears that the 76S mutation is not involved in ex vivo quinoline reduced susceptibility, and particularly in reduced susceptibility to artemisinin derivative partners in artemisinin-based combination therapy (ACT), including amodiaquine, lumefantrine, piperaquine, pyronaridine, or mefloquine. A previous study showed that the 76S mutation was observed in 31.3% of parasites collected from patients who continued to be parasitaemic on day 3 after ACT treatment and in 15.3% of isolates from patients who were successfully cured in Senegal [17]. However, the difference was not significant (*p* = 0.151).

## 5. Conclusions

The three mutations in the *pfcoronin* gene which are responsible for artemisinin resistance were not reported in this study. Current data showed that the 76S mutation is present in many countries of West Africa (mean prevalence of 20.7%) and Central Africa (mean prevalence of 11.9%). This mutation does not seem to be predictive of in vitro reduced susceptibility to quinolines, including artemisinin derivative partners in ACT such as amodiaquine, lumefantrine, piperaquine, pyronaridine, or mefloquine. It is reassuring that this mutation does not confer in vitro resistance to ACT partners. One limitation of this study was that it did not assess in vitro susceptibility to artemisinin using the ring survival assay (RSA) to determine the association between in vitro reduced susceptibility to artemisinin and the 76S mutation. These data do not allow the potential involvement of mutations in the *pfcoronin* gene in artemisinin resistance to be discounted.

## Figures and Tables

**Table 1:** Proportion of mutations in the *pfcoronin* gene according to the geographical origin of the *Plasmodium falciparum* isolates.

Areas	Isolates No and (%)	P76S Mutation No and % ^a^	V62M Mutation No and % ^a^
West Africa	357 (40.8%)	74 (8.5%)	3 (0.3%)
Central Africa	335 (38.3%)	40 (4.5%)	6 (0.7%)
East Africa	143 (16.3%)	6 (0.7%)	0
Unspecified country in Africa	39 (4.6%)	5 (0.6%)	0
Total	874	125 (14.3%)	9 (1.0%)

^a^ Proportion of 76S and 62M mutations compared to the total number of isolates from Africa.

**Table 2:** Proportion of *pfcoronin* 76S and 62M mutations in *Plasmodium falciparum* isolates per country and per year.

Country	2018 No and %		2019 No and %		2018–2019 No and %	
	76S Mutation	62M Mutation	76S Mutation	62M Mutation	76S Mutation	62M Mutation
**West Africa**	**38/179 (21.2)**	**2/179 (1.1)**	**36/178 (20.2)**	**1/178 (0.6)**	**74/357 (20.7)**	**3/357 (0.8)**
Côte d’Ivoire	20/72 (27.8)	0/20	14/69 (20.3)	0/69	34/141 (24.1)	0/141
Guinea	4/25 (16.0)	0/25	6/29 (20.7)	0/29	10/54 (18.5)	0/54
Togo	1/14 (7.1)	0/14	2/20 (10.0)	0/20	3/34 (8.8)	0/34
Burkina Faso	4/19 (21.1)	2/19 (10.5)	2/10 (20.0)	0/10	6/29 (20.7)	2/29 (6.9)
Benin	2/13 (15.4)	0/13	4/12 (33.3)	0/12	6/25 (24.0)	0/25
Mali	2/8 (25.0)	0/8	2/8 (25.0)	0/8	4/16 (25.0)	0/16
Nigeria	2/5 (40.0)	0/5	2/12 (16.7)	1/12 (8.3)	4/17 (23.5)	1/17 (5.9)
Senegal	1/8 (12.5)	0/8	0/3	0/3	1/11 (9.1)	0/11
Niger	0/3	0/3	3/9 (33.3)	0/9	3/12 (25.0)	0/12
Ghana	1/4 (25.0)	0/4	1/5 (20.0)	0/5	2/9 (22.2)	0/9
Sierra Leone	1/4 (25.0)	0/4	0	0	1/4 (25.0)	0/4
Guinea Conakry	0/3	0/3	0/1	0/1	0/4	0/4
Cape Verde	0/1	0/1	0	0	0/1	0/1
**Central Africa**	**20/154 (13.0)**	**1/154 (0.6)**	**20/181 (11.0)**	**5/181 (2.8)**	**40/335 (11.9)**	**6/335 (1.8)**
Cameroon	8/68 (11.8)	0/68	11/79 (13.9)	2/79 (2.5)	19/147 (12.9)	2/147 (1.4)
Gabon	3/28 (10.7)	0/28	2/28 (7.1)	1/28 (3.6)	5/56 (8.9)	1/56 (1.8)
Democratic Republic of Congo	2/22 (9.1)	0/22	1/28 (3.6)	0/28	3/50 (6.0)	0/50
Central African Republic	6/19 (3.2)	0/19	4/31 (12.9)	2/31 (6.5)	10/50 (20.0)	2/50 (4.0)
Chad	1/13 (7.7)	1/13 (7.7)	2/13 (15.4)	0/13	3/26 (11.5)	2/26 (3.8)
Angola	0/2	0/2	0/1	0/1	0/3	0/3
Equatorial Guinea	0/2	0/2	0/1	0/1	0/3	0/3
**East Africa**	**1/56 (1.8)**	**0/56**	**5/87 (5.7)**	**0/87 (0.0)**	**6/143 (4.2)**	**0/143 (0.0)**
Mayotte	1/3 (33.3)	0/3	1/2 (50.0)	0/2	2/5 (40.0)	0/5
Eritrea	0	0	1/1 (100.0)	0/1	1/1 (100.0)	0/1
Tanzania	0/1	0/1	2/7 (28.6)	0/7	2/8 (25.0)	0/8
Sudan	0	0	1/2 (50.0)	0/2	1/2 (50.0)	0/2
Kenya	0	0	0/1	0/1	0/1	0/1
Zambia	0	0	0/1	0/1	0/1	0/1
Uganda	0	0	0/2	0/2	0/2	0/2
Mozambique	0/1	0/1	0	0	0/1	0/1
Djibouti	0/6	0/6	0/6	0/6	0/12	0/12
Comoros	0/40	0/40	0/64	0/64	0/104	0/104
Madagascar	0/5	0/5	0/1	0/1	0/6	0/6
**Unspecified African country**	**2/8 (25.0)**	**0/8**	**3/31 (9.7)**	**0/31 (0.0)**	**5/39 (12.8)**	**0/39 (0.0)**
Total	31/397 (15.4)	3/397 (0.8)	64/477 (13.4)	6/477 (1.3)	128/874 (14.3)	9/874 (1.0)

Bold represents the African area to which the countries listed below belong.

**Table 3:** Ex vivo susceptibility geometric mean to chloroquine, quinine, lumefantrine, desethylamodiaquine, pyronaridine, and piperaquine according to the *pfcoronin* genotype (P76 or 76S).

Drug (Isolates No)	Isolates Wild-Type (P76)		Isolates with 76S Mutation		*p*-Value (*t*-Test)
	IC_50_ Geometric Mean (nM)	Min-Max IC_50_	IC_50_ Geometric Mean (nM)	Min-Max IC_50_	
Chloroquine (328)	84.0	2.8–764.7	95.2	8.0–350.9	0.463
Quinine (325)	281.0	6.2–757.2	268.7	23.3–664.7	0.617
Lumefantrine (329)	6.7	0.4–68.8	6.5	0.5–46.0	0.878
Desethylamodiaquine (306)	40.7	0.6–775.9	34.3	2.0–152.5	0.343
Mefloquine (321)	22.9	0.7–98.0	26.1	3.2–87.8	0.295
Pyronaridine (319)	14.0	0.2–119.3	12.6	1.0–56.9	0.410
Piperaquine (321)	25.1	5.5–67.1	25.2	0.7–379.3	0.983

**Table 4:** Proportions of isolates with in vitro reduced susceptibility to chloroquine, quinine, lumefantrine, desethylamodiaquine, pyronaridine, and piperaquine according to the *pfcoronin* genotype (P76 or 76S).

Drug (No Isolates)	% of Isolates with Reduced Susceptibility (No of Isolates with Reduced Susceptibility/Total of Isolates)		*p*-Value (Chi-Squared Test)
	Wild-Type Isolates (P76)	Isolates with 76S Mutation	
Chloroquine (328)	25.1 (70/279)	32.7 (16/49)	0.506
Quinine (325)	0 (0/276)	0 (0/49)	0
Lumefantrine (329)	0 (0/280)	0 (0/49)	0
Desethylamodiaquine (306)	8.0 (21/262)	9.1 (4/44)	1
Mefloquine (321)	23.4 (64/273)	29.2 (14/48)	0.631
Pyronaridine (319)	1.1 (3/273)	0 (0/46)	1
Piperaquine (321)	0.7 (2/273)	0 (0/48)	0.999

## Data Availability

The data presented in this study are available on request from the corresponding author.
